# Supplementary material for: The association of skin autofluorescence with cardiovascular events and all-cause mortality in persons with chronic kidney disease stage 3: A prospective cohort study
Source: PLoS Med. 2020 Jul 13;17(7):e1003163. doi: 10.1371/journal.pmed.1003163 (PMC7357739; doi:10.1371/journal.pmed.1003163)
Supplement: S1 Table — CVE, cardiovascular event. (DOCX) [file pmed.1003163.s001.docx]

**S1 Table:** Cox Proportional Hazards model showing variables associated with time to non-fatal cardiovascular events.

| Variable | Univariable | | Model 1 (n=1705) | | Model 2 (n=1701) |  | Model 3 (n=1673) |  |
| --- | --- | --- | --- | --- | --- | --- | --- | --- |
|  | HR (95% CI) | p-value | HR (95% CI) | p-value | HR (95% CI) | p-value | HR (95% CI) | p-value |
| SAF | 1.32 (1.21 to 1.44) | <0.001 | 1.19 (1.08 to 1.30) | <0.001 | 1.15 (1.05 to 1.27) | 0.004 | 1.12 (1.01 to 1.23) | 0.03 |
| Age | 1.43 (1.29 to 1.57) | <0.001 | 1.30 (1.17 to 1.44) | <0.001 | 1.28 (1.14 to 1.43) | <0.001 | 1.27 (1.13 to 1.42) | <0.001 |
| Male sex | 1.61 (1.35 to 1.92) | <0.001 | 1.36 (1.13 to 1.63) | 0.001 | 1.37 (1.13 to 1.66) | 0.001 | 1.44 (1.15 to 1.81) | 0.001 |
| Diabetes | 1.24 (0.99 to 1.56) | 0.06 | 1.04 (0.83 to 1.32) | 0.7 | 0.90 (0.71 to 1.15) | 0.4 | 0.91 (0.70 to 1.17) | 0.5 |
| Previous CVD | 2.23 (1.84 to 2.69) | <0.001 | 1.79 (1.47 to 2.17) | <0.001 | 1.76 (1.45 to 2.15) | <0.001 | 1.79 (1.47 to 2.20) | <0.001 |
| Hypertension | 1.60 (1.17 to 2.18) | 0.003 | 1.24 (0.90 to 1.70) | 0.2 | 1.12 (0.80 to 1.55) | 0.5 | 1.16 (0.83 to 1.62) | 0.4 |
| Ever smoked | 1.37 (1.14 to 1.64) | 0.001 | 1.13 (0.93 to 1.36) | 0.2 | 1.10 (0.91 to 1.33) | 0.3 | 1.08 (0.89 to 1.31) | 0.4 |
| Systolic BP | 1.06 (0.97 to 1.16) | 0.2 |  |  | 1.01 (0.90 to 1.13) | 0.9 | 1.02 (0.91 to 1.14) | 0.8 |
| Diastolic BP | 0.83 (0.76 to 0.91) | <0.001 |  |  | 0.91 (0.81 to 1.02) | 0.09 | 0.92 (0.81 to 1.03) | 0.1 |
| BMI | 1.07 (0.99 to 1.17) | 0.1 |  |  | 1.16 (1.06 to 1.27) | 0.002 | 1.12 (1.02 to 1.24) | 0.02 |
| eGFR | 0.76 (0.69 to 0.83) | <0.001 |  |  | 0.94 (0.85 to 1.05) | 0.3 | 1.00 (0.89 to 1.12) | 0.95 |
| UACR (log) | 1.25 (1.14 to 1.37) | <0.001 |  |  | 1.13 (1.03 to 1.25) | 0.01 | 1.10 (1.00 to 1.21) | 0.06 |
| Albumin | 0.80 (0.74 to 0.87) | <0.001 |  |  |  |  | 0.89 (0.81 to 0.98) | 0.01 |
| Uric acid | 1.19 (1.09 to 1.30) | <0.001 |  |  |  |  | 1.02 (0.92 to 1.14) | 0.7 |
| Total cholesterol | 0.84 (0.76 to 0.92) | <0.001 |  |  |  |  | 1.04 (0.93 to 1.15) | 0.5 |
| HDL cholesterol | 0.82 (0.75 to 0.90) | <0.001 |  |  |  |  | 0.92 (0.83 to 1.03) | 0.2 |
| Haemoglobin | 0.85 (0.77 to 0.93) | <0.001 |  |  |  |  | 0.92 (0.83 to 1.02) | 0.1 |
| hsCRP (log) | 1.25 (1.15 to 1.36) | <0.001 |  |  |  |  | 1.12 (1.01 to 1.23) | 0.03 |

Hazard ratios for continuous variables are expressed per standard deviation (SD) change

Abbreviations: BMI – body mass index, BP – blood pressure, CI – confidence interval, CVD – cardiovascular disease, eGFR - estimated glomerular filtration rate, HDL – high density lipoprotein, HR – hazard ratio, hsCRP – high sensitivity C reactive protein, SAF - Skin autofluorescence, UACR - urine albumin to creatinine ratio.
