# Supplementary material for: The association of skin autofluorescence with cardiovascular events and all-cause mortality in persons with chronic kidney disease stage 3: A prospective cohort study
Source: PLoS Med. 2020 Jul 13;17(7):e1003163. doi: 10.1371/journal.pmed.1003163 (PMC7357739; doi:10.1371/journal.pmed.1003163)
Supplement: S2 Table — CVE, cardiovascular event. (DOCX) [file pmed.1003163.s002.docx]

**S2 Table:** Cox Proportional Hazards model showing variables associated with time to fatal cardiovascular events.

| Variable | Univariable | | Model 1 (n=1707) | | Model 2 (n=1704) |  | Model 3 (n=1681) |  |
| --- | --- | --- | --- | --- | --- | --- | --- | --- |
|  | HR (95% CI) | p-value | HR (95% CI) | p-value | HR (95% CI) | p-value | HR (95% CI) | p-value |
| SAF | 1.53 (1.29 to 1.82) | <0.001 | 1.24 (1.03 to 1.48) | 0.02 | 1.13 (0.94 to 1.37) | 0.2 | 1.12 (0.92 to 1.35) | 0.3 |
| Age | 2.41 (1.89 to 3.07) | <0.001 | 2.12 (1.65 to 2.73) | <0.001 | 1.67 (1.29 to 2.16) | <0.001 | 1.68 (1.29 to 2.18) | <0.001 |
| Male sex | 2.55 (1.72 to 3.77) | <0.001 | 1.82 (1.21 to 2.74) | 0.004 | 1.75 (1.17 to 2.63) | 0.007 | 1.76 (1.15 to 2.69) | 0.009 |
| Diabetes | 1.54 (0.98 to 2.43) | 0.06 |  |  |  |  |  |  |
| Previous CVD | 4.13 (2.82 to 6.06) | <0.001 | 2.86 (1.94 to 4.23) | <0.001 | 2.81 (1.90 to 4.16) | <0.001 | 2.88 (1.93 to 4.30) | <0.001 |
| Hypertension | 1.77 (0.86 to 3.63) | 0.1 |  |  |  |  |  |  |
| Ever smoked | 1.92 (1.27 to 2.90) | 0.002 | 1.32 (0.86 to 2.03) | 0.2 |  |  |  |  |
| Systolic BP | 0.99 (0.82 to 1.21) | 0.9 |  |  |  |  |  |  |
| Diastolic BP | 0.66 (0.54 to 0.81) | <0.001 |  |  | 0.83 (0.67 to 1.03) | 0.08 |  |  |
| BMI | 0.84 (0.68 to 1.03) | 0.09 |  |  |  |  |  |  |
| eGFR | 0.44 (0.36 to 0.54) | <0.001 |  |  | 0.60 (0.48 to 0.77) | <0.001 | 0.57 (0.44 to 0.74) | <0.001 |
| UACR (log) | 1.60 (1.29 to 1.98) | <0.001 |  |  | 1.21 (0.97 to 1.51) | 0.09 | 1.15 (0.92 to 1.44) | 0.2 |
| Albumin | 0.77 (0.66 to 0.91) | 0.002 |  |  |  |  | 0.85 (0.70 to 1.03) | 0.1 |
| Uric acid | 1.31 (1.08 to 1.57) | 0.005 |  |  |  |  | 0.91 (0.74 to 1.12) | 0.4 |
| Total cholesterol | 0.64 (0.52 to 0.80) | <0.001 |  |  |  |  | 0.94 (0.74 to 1.18) | 0.6 |
| HDL cholesterol | 0.72 (0.58 to 0.89) | 0.003 |  |  |  |  |  |  |
| Haemoglobin | 0.83 (0.68 to 1.00) | 0.06 |  |  |  |  |  |  |
| hsCRP (log) | 1.26 (1.05 to 1.51) | 0.02 |  |  |  |  | 1.05 (0.86 to 1.28) | 0.6 |

Hazard ratios for continuous variables are expressed per standard deviation (SD) change

Abbreviations: BMI – body mass index, BP – blood pressure, CI – confidence interval, CVD – cardiovascular disease, eGFR - estimated glomerular filtration rate, HDL – high density lipoprotein, HR – hazard ratio, hsCRP – high sensitivity C reactive protein, SAF - Skin autofluorescence, UACR - urine albumin to creatinine ratio.
