# Supplementary material for: The association of skin autofluorescence with cardiovascular events and all-cause mortality in persons with chronic kidney disease stage 3: A prospective cohort study
Source: PLoS Med. 2020 Jul 13;17(7):e1003163. doi: 10.1371/journal.pmed.1003163 (PMC7357739; doi:10.1371/journal.pmed.1003163)
Supplement: S3 Table — CVE, cardiovascular event; SAF, skin autofluorescence. (DOCX) [file pmed.1003163.s003.docx]

**S3 Table:** Cox Proportional Hazards model showing independent associations with time to first cardiovascular event in the subgroup of participants who had follow-up assessment of skin autofluorescence at Year 1 and no CVE prior to Year 1.

| Variable | Multivariable (n=1508) | |
| --- | --- | --- |
|  | HR (95% CI) | p-value |
| SAF | 1.12 (1.00 to 1.27) | 0.06 |
| Age | 1.28 (1.13 to 1.46) | <0.001 |
| Male sex | 1.63 (1.28 to 2.08) | <0.001 |
| Diabetes | 0.96 (0.72 to 1.26) | 0.8 |
| Previous CVD | 1.83 (1.47 to 2.27) | <0.001 |
| Hypertension | 1.20 (0.82 to 1.77) | 0.4 |
| Ever smoked | 1.07 (0.87 to 1.32) | 0.5 |
| SBP | 1.05 (0.93 to 1.19) | 0.4 |
| DBP | 0.85 (0.74 to 0.96) | 0.01 |
| BMI | 1.13 (1.01 to 1.26) | 0.03 |
| eGFR | 0.92 (0.81 to 1.05) | 0.2 |
| UACR (log) | 1.12 (1.01 to 1.25) | 0.04 |
| Albumin | 0.93 (0.84 to 1.03) | 0.1 |
| Uric acid | 1.00 (0.89 to 1.12) | 1.0 |
| Total cholesterol | 1.08 (0.97 to 1.21) | 0.2 |
| HDL cholesterol | 0.92 (0.81 to 1.04) | 0.2 |
| Haemoglobin | 0.96 (0.86 to 1.08) | 0.5 |
| hsCRP (log) | 1.07 (0.96 to 1.19) | 0.2 |
| Delta SAF | 1.11 (1.00 to 1.22) | 0.04 |

Hazard ratios for continuous variables are expressed per standard deviation (SD) change

Abbreviations: BMI – body mass index, BP – blood pressure, CI – confidence interval, CVD – cardiovascular disease, eGFR - estimated glomerular filtration rate, HDL – high density lipoprotein, HR – hazard ratio, hsCRP – high sensitivity C reactive protein, SAF - Skin autofluorescence, UACR - urine albumin to creatinine ratio.
