# Supplementary material for: The association of skin autofluorescence with cardiovascular events and all-cause mortality in persons with chronic kidney disease stage 3: A prospective cohort study
Source: PLoS Med. 2020 Jul 13;17(7):e1003163. doi: 10.1371/journal.pmed.1003163 (PMC7357739; doi:10.1371/journal.pmed.1003163)
Supplement: S4 Table — (DOCX) [file pmed.1003163.s004.docx]

**S4 Table:** Cox Proportional Hazards model showing variables associated with time to death due to non-cardiovascular causes.

| Variable | Univariable | | Model 1 (n=1705) | | Model 2 (n=1701) |  | Model 3 (n=1673) |  |
| --- | --- | --- | --- | --- | --- | --- | --- | --- |
|  | HR (95% CI) | p-value | HR (95% CI) | p-value | HR (95% CI) | p-value | HR (95% CI) | p-value |
| SAF | 1.50 (1.33 to 1.70) | <0.001 | 1.27 (1.11 to 1.45) | <0.001 | 1.22 (1.06 to 1.40) | 0.005 | 1.18 (1.03 to 1.36) | 0.02 |
| Age | 2.48 (2.09 to 2.94) | <0.001 | 2.32 (1.95 to 2.77) | <0.001 | 2.07 (1.71 to 2.50) | <0.001 | 2.04 (1.68 to 2.48) | <0.001 |
| Male sex | 1.80 (1.38 to 2.36) | <0.001 | 1.38 (1.04 to 1.83) | 0.03 | 1.24 (0.93 to 1.65) | 0.2 | 1.27 (0.90 to 1.79) | 0.2 |
| Diabetes | 1.40 (1.01 to 1.95) | 0.05 | 1.16 (0.83 to 1.63) | 0.4 | 1.10 (0.77 to 1.56) | 0.6 | 1.09 (0.75 to 1.58) | 0.7 |
| Previous CVD | 1.76 (1.32 to 2.36) | <0.001 | 1.24 (0.92 to 1.67) | 0.2 | 1.23 (0.91 to 1.66) | 0.2 | 1.20 (0.88 to 1.64) | 0.2 |
| Hypertension | 1.61 (0.99 to 2.61) | 0.05 | 1.02 (0.62 to 1.67) | 0.9 | 0.90 (0.54 to 1.50) | 0.7 | 0.88 (0.52 to 1.47) | 0.6 |
| Ever smoked | 1.62 (1.23 to 2.15) | 0.001 | 1.34 (1.00 to 1.79) | 0.05 | 1.28 (0.95 to 1.72) | 0.1 | 1.19 (0.88 to 1.60) | 0.3 |
| Systolic BP | 1.18 (1.03 to 1.35) | 0.01 |  |  | 1.03 (0.87 to 1.20) | 0.8 | 1.04 (0.88 to 1.23) | 0.6 |
| Diastolic BP | 0.80 (0.70 to 0.92) | 0.002 |  |  | 0.98 (0.82 to 1.15) | 0.8 | 1.00 (0.84 to 1.19) | 0.99 |
| BMI | 0.85 (0.74 to 0.98) | 0.03 |  |  | 0.94 (0.80 to 1.10) | 0.4 | 0.90 (0.76 to 1.06) | 0.2 |
| eGFR | 0.56 (0.49 to 0.64) | <0.001 |  |  | 0.80 (0.68 to 0.94) | 0.007 | 0.88 (0.73 to 1.05) | 0.2 |
| UACR (log) | 1.42 (1.23 to 1.64) | <0.001 |  |  | 1.19 (1.02 to 1.38) | 0.03 | 1.09 (0.94 to 1.28) | 0.3 |
| Albumin | 0.78 (0.70 to 0.87) | <0.001 |  |  |  |  | 0.97 (0.83 to 1.12) | 0.6 |
| Uric acid | 1.30 (1.14 to 1.48) | <0.001 |  |  |  |  | 1.10 (0.94 to 1.28) | 0.3 |
| Total cholesterol | 0.74 (0.64 to 0.86) | <0.001 |  |  |  |  | 0.92 (0.77 to 1.09) | 0.3 |
| HDL cholesterol | 0.85 (0.74 to 0.99) | 0.03 |  |  |  |  | 1.01 (0.86 to 1.19) | 0.9 |
| Haemoglobin | 0.73 (0.64 to 0.83) | <0.001 |  |  |  |  | 0.90 (0.78 to 1.05) | 0.2 |
| hsCRP (log) | 1.49 (1.32 to 1.68) | <0.001 |  |  |  |  | 1.34 (1.17 to 1.54) | <0.001 |

Hazard ratios for continuous variables are expressed per standard deviation (SD) change

Abbreviations: BMI – body mass index, BP – blood pressure, CI – confidence interval, CVD – cardiovascular disease, eGFR - estimated glomerular filtration rate, HDL – high density lipoprotein, HR – hazard ratio, hsCRP – high sensitivity C reactive protein, SAF - Skin autofluorescence, UACR - urine albumin to creatinine ratio.
