# Supplementary material for: The association of skin autofluorescence with cardiovascular events and all-cause mortality in persons with chronic kidney disease stage 3: A prospective cohort study
Source: PLoS Med. 2020 Jul 13;17(7):e1003163. doi: 10.1371/journal.pmed.1003163 (PMC7357739; doi:10.1371/journal.pmed.1003163)
Supplement: S5 Table — SAF, skin autofluorescence. (DOCX) [file pmed.1003163.s005.docx]

**S5 Table:** Cox Proportional Hazards model showing independent associations with time to death from any cause in the subgroup participants who had follow-up assessment of skin autofluorescence at Year 1 (n=1567).

| Variable | Multivariable | |
| --- | --- | --- |
|  | HR (95% CI) | p-value |
| SAF | 1.25 (1.07 to 1.45) | 0.005 |
| Age | 2.00 (1.66 to 2.42) | <0.001 |
| Male sex | 1.43 (1.03 to 1.99) | 0.03 |
| Diabetes | 1.01 (0.70 to 1.45) | 1.0 |
| Previous CVD | 1.43 (1.07 to 1.90) | 0.02 |
| Hypertension | 0.92 (0.55 to 1.53) | 0.7 |
| Ever smoked | 1.17 (0.88 to 1.57) | 0.3 |
| SBP | 1.02 (0.87 to 1.21) | 0.8 |
| DBP | 0.94 (0.79 to 1.12) | 0.5 |
| BMI | 0.93 (0.79 to 1.09) | 0.4 |
| eGFR | 0.75 (0.62 to 0.89) | 0.001 |
| UACR (log) | 1.08 (0.92 to 1.25) | 0.4 |
| Albumin | 0.99 (0.85 to 1.14) | 0.9 |
| Uric acid | 0.95 (0.82 to 1.10) | 0.5 |
| Total cholesterol | 0.94 (0.80 to 1.11) | 0.5 |
| HDL cholesterol | 0.95 (0.80 to 1.12) | 0.6 |
| Haemoglobin | 0.95 (0.82 to 1.10) | 0.5 |
| hsCRP (log) | 1.21 (1.05 to1.38) | 0.007 |
| Delta SAF | 1.24 (1.09 to 1.41) | 0.001 |

Hazard ratios for continuous variables are expressed per standard deviation (SD) change

Abbreviations: BMI – body mass index, BP – blood pressure, CI – confidence interval, CVD – cardiovascular disease, eGFR - estimated glomerular filtration rate, HDL – high density lipoprotein, HR – hazard ratio, hsCRP – high sensitivity C reactive protein, SAF - Skin autofluorescence, UACR - urine albumin to creatinine ratio.
