# Supplementary material for: The association of skin autofluorescence with cardiovascular events and all-cause mortality in persons with chronic kidney disease stage 3: A prospective cohort study
Source: PLoS Med. 2020 Jul 13;17(7):e1003163. doi: 10.1371/journal.pmed.1003163 (PMC7357739; doi:10.1371/journal.pmed.1003163)
Supplement: S6 Table — CVE, cardiovascular event. (DOCX) [file pmed.1003163.s006.docx]

**S6 Table:** Cox Proportional Hazards model showing independent determinants of time to first cardiovascular event in the subgroup participants without Diabetes Mellitus at baseline (n=1423).

| Variable | Multivariable | |
| --- | --- | --- |
|  | HR (95% CI) | p-value |
| SAF | 1.13 (1.02 to 1.25) | 0.02 |
| Age | 1.30 (1.15 to 1.46) | <0.001 |
| Male sex | 1.56 (1.23 to 1.98) | <0.001 |
| Previous CVD | 1.74 (1.41 to 2.15) | <0.001 |
| Hypertension | 1.29 (0.93 to 1.80) | 0.1 |
| Ever smoked | 1.00 (0.82 to 1.23) | 1.0 |
| SBP | 1.00 (0.89 to 1.13) | 1.0 |
| DBP | 0.88 (0.78 to 1.00) | 0.05 |
| BMI | 1.06 (0.95 to 1.18) | 0.3 |
| eGFR | 0.89 (0.79 to 1.00) | 0.05 |
| UACR (log) | 1.09 (0.99 to 1.20) | 0.1 |
| Albumin | 0.89 (0.81 to 0.98) | 0.02 |
| Uric acid | 0.97 (0.87 to 1.08) | 0.6 |
| Total cholesterol | 1.00 (0.90 to 1.11) | 1.0 |
| HDL cholesterol | 0.92 \|(0.82 to 1.03) | 0.1 |
| Haemoglobin | 0.97 (0.87 to 1.07) | 0.5 |
| hsCRP (log) | 1.13 (1.02 to 1.25) | 0.02 |

Hazard ratios for continuous variables are expressed per standard deviation (SD) change

Abbreviations: BMI – body mass index, BP – blood pressure, CI – confidence interval, CVD – cardiovascular disease, eGFR - estimated glomerular filtration rate, HDL – high density lipoprotein, HR – hazard ratio, hsCRP – high sensitivity C reactive protein, SAF - Skin autofluorescence, UACR - urine albumin to creatinine ratio.
