# Supplementary material for: The association of skin autofluorescence with cardiovascular events and all-cause mortality in persons with chronic kidney disease stage 3: A prospective cohort study
Source: PLoS Med. 2020 Jul 13;17(7):e1003163. doi: 10.1371/journal.pmed.1003163 (PMC7357739; doi:10.1371/journal.pmed.1003163)
Supplement: S7 Table — (DOCX) [file pmed.1003163.s007.docx]

**S7 Table:** Cox Proportional Hazards model showing independent determinants of time to death from any cause in the subgroup participants without Diabetes Mellitus at baseline (n=1423).

| Variable | Multivariable | |
| --- | --- | --- |
|  | HR (95% CI) | p-value |
| SAF | 1.08 (0.95 to 1.24) | 0.3 |
| Age | 2.16 (1.79 to 2.61) | <0.001 |
| Male sex | 1.22 (0.88 to 1.69) | 0.2 |
| Previous CVD | 1.59 (1.21 to 2.10) | 0.001 |
| Hypertension | 1.02 (0.64 to 1.62) | 0.9 |
| Ever smoked | 1.22 (0.92 to 1.62) | 0.2 |
| SBP | 0.98 (0.84 to 1.14) | 0.8 |
| DBP | 0.94 (0.80 to 1.10) | 0.4 |
| BMI | 0.87 (0.75 to 1.03) | 0.1 |
| eGFR | 0.75 (0.63 to 0.89) | 0.001 |
| UACR (log) | 1.05 (0.91 to 1.20) | 0.5 |
| Albumin | 0.91 (0.79 to 1.05) | 0.2 |
| Uric acid | 1.01 (0.87 to 1.17) | 0.9 |
| Total cholesterol | 0.88 (0.75 to 1.03) | 0.1 |
| HDL cholesterol | 0.97 (0.83 to 1.14) | 0.7 |
| Haemoglobin | 1.01 (0.88 to 1.17) | 0.9 |
| hsCRP(log) | 1.31 (1.15 to 1.50) | <0.001 |

Hazard ratios for continuous variables are expressed per standard deviation (SD) change

Abbreviations: BMI – body mass index, BP – blood pressure, CI – confidence interval, CVD – cardiovascular disease, eGFR - estimated glomerular filtration rate, HDL – high density lipoprotein, HR – hazard ratio, hsCRP – high sensitivity C reactive protein, SAF - Skin autofluorescence, UACR - urine albumin to creatinine ratio
