# Supplementary material for: The association of skin autofluorescence with cardiovascular events and all-cause mortality in persons with chronic kidney disease stage 3: A prospective cohort study
Source: PLoS Med. 2020 Jul 13;17(7):e1003163. doi: 10.1371/journal.pmed.1003163 (PMC7357739; doi:10.1371/journal.pmed.1003163)
Supplement: S1 Protocol — (DOCX) [file pmed.1003163.s009.docx]

**DEFINING THE RISK OF KIDNEY FUNCTION DECLINE AND CARDIOVASCULAR DISEASE AMONG PATIENTS WITH CHRONIC KIDNEY DISEASE STAGE 3: THE RENAL RISK IN DERBY (R^2^ID) STUDY**

**Investigator**

Dr M.W. Taal

Department of Renal Medicine, Royal Derby Hospital, Uttoxeter Road, Derby DE22 3NE

Tel 01332 789344 Fax 01332 789352

e-mail: maarten.taal1@nhs.net

MBChB, MMed, MD, FCP(SA), FRCP.

Consultant Renal Physician

**Funders**

Dunhill Medical Trust

3^rd^ Floor 16-18 Marshalsea Road, London, SE1 1HL

**Research Team Members**

Dr A. Shardlow Research Fellow

Dr R.J. Fluck Consultant Renal Physician

Prof C.W. McIntyre Professor of Nephrology and Honorary Consultant Renal Physician

Department of Renal Medicine

Tel 01332 789344

**Sponsor**

Derby Hospitals NHS Foundation Trust

**Trust(s) where research will take place**

Derby Hospitals NHS Foundation Trust

In order to comply with the Research Governance Framework “a senior individual with appropriate experience must be designated as the Chief Investigator/Investigator for any research undertaken in or through the NHS or social services or using patient’s organs, tissue or data” The investigator must have **suitable experience** and be willing to oversee the study in accordance with principal investigator responsibilities as stated in the Research Governance Framework 3.6.3.

For research being undertaken by all junior and research inexperienced members of staff in Derby Hospitals NHS Foundation Trust, an Investigator must be identified and asked if they willing to take on the role of Principal Investigator.

| **Date submitted to R&D Office:** |  |
| --- | --- |
| **Protocol Number:** | **RD-5103-024-07** |

**Table of Contents**

Study Summary 3

1. Introduction 4

1.1 Background 4

2 Study Objectives 5

3 Study Design 5

3.1 General Design 5

3.2 Primary Study Endpoints 6

3.3 Secondary Study Endpoints 6

4 Methods 6

4.1 Subject Selection and Withdrawal 6

4.2 Subject Recruitment and Screening 7

4.3 Withdrawal of Subjects 7

4.3.1 When and How to Withdraw Subjects 7

4.3.2 Data Collection and Follow-up for Withdrawn Subjects 8

4.4 Prior and Concomitant Therapy 8

5 Laboratory Assays 8

6 Randomisation 9

7 Study Procedures 9

8 Statistical Plan 11

8.1 Sample Size Determination 11

8.2 Statistical Methods 11

8.3 Subject Population(s) for Analysis 12

9 Safety and Adverse Events 12

9.1 Recording of Adverse Events 12

9.2 When Adverse Events are Recorded 12

10 Data Handling and Record Keeping 13

10.1 Confidentiality 13

10.2 Source Documents 14

10.3 Case Report Forms 14

10.4 Records Retention 15

11 Study Monitoring, Auditing, and Inspecting 15

11.1 Study Monitoring Plan 15

12 Ethical Considerations 15

13 Study Finances 16

13.1 Funding Source 16

13.2 Indemnity for the performance of the study 16

13.3 Sponsorship: 16

14 Publication Plan 17

15 References 17

16 Attachments 19

# Study Summary

# 1. Introduction

**Title**

Defining the risk of renal function decline and cardiovascular disease among patients with chronic kidney disease stage 3: The Renal Risk in Derby (R^2^ID) Study.

**Protocol Version Number and Date**

Version 2.3

October 2013

**Short Title**

Renal and Cardiovascular Risk in CKD stage 3

**Methodology**

Long term observational study to assess risk factors.

**Study Duration**

Eleven years and 10 months. See section 3.1 for details.

**Study Centres**

Royal Derby Hospital

GP Practices within Southern Derbyshire CCG, North Derbyshire CCG

and Erewash CCG.

**Objectives**

1. To define the risk of kidney function decline in a cohort of patients with chronic kidney disease (CKD) stage 3.
2. To define the risk of cardiovascular disease in a cohort of patients with chronic kidney disease (CKD) stage 3.

**Number of Subjects/Patients**

1822

**Main Inclusion Criteria**

1. CKD stage 3 as defined by the K/DOQI classification (i.e. GFR 30-59ml/min/1.73m^2^ on 2 occasions at least 3 months apart)
2. Age >18 years

**Statistical Methodology and Analysis**

Descriptive statistics for baseline data.

Univariate and Multivariate analysis to investigate associations between CKD, cardiovascular disease and risk factors at baseline.

Cox regression analysis to investigate risk factors for CKD progression and cardiovascular events.

The described research study will be conducted in compliance with the protocol, the Research Governance Framework, International Conference on Harmonisation Good Clinical Practice Guideline ICH/GCP, and all applicable Derby Hospitals NHS Foundation Trust Research Office requirements.

## Background

The introduction of estimated glomerular filtration rate (GFR) to assess renal function has identified large numbers of patients with previously undetected chronic kidney disease (CKD). It is clear from previous studies that there is considerable heterogeneity in the risk of decline in renal function among patients with similar levels of GFR. Patients therefore differ widely in their need for specialist referral and treatment. Epidemiological studies have identified multiple risk factors for CKD progression [1] but few have sought to define the risk in individual patients. Several attempts have been made to develop scoring systems to predict renal risk but these studies have largely been limited to patients with specific kidney diseases and have not been validated in more general populations of patients with CKD [2-4]. Chronic kidney disease is also associated with an increased risk of cardiovascular disease that in many cases’ exceeds the risk of developing end-stage renal disease (ESRD) [5]. It is not known, however, whether traditional cardiovascular risk factors are relevant in patients with CKD or whether cardiovascular risk assessment tools used in the general population are applicable to patients with CKD. Recent data indicates that the extent of advanced glycation end product (AGE) deposition in the skin is an important marker of cardiovascular risk among patients with CKD [6] and periodontal status has also been associated with risk of cardiovascular disease in those with and without CKD [7-10]. Preliminary data suggest that cardiovascular disease may also be a risk factor for CKD progression. Thus kidney and cardiovascular disease may exacerbate each other, leading to a vicious circle of progressive cardiovascular and renal decline.

# Study Objectives

## To define the risk of kidney function decline in a cohort of patients with chronic kidney disease (CKD) stage 3.

## To define the risk of cardiovascular disease in a cohort of patients with CKD stage 3.

## To develop a comprehensive description of patients with CKD stage 3, who are currently followed up by General Practitioners.

## To assess the management of patients with CKD stage 3 by General Practitioners compared with Renal Association Clinical Practice Guidelines.

## To develop care plans to improve the care of patients with CKD stage 3 within Primary Care.

## To assess the value of monitoring urine albumin to creatinine ratio (UACR), urine sodium and urine phosphate in the management of patients with CKD stage 3.

## To assess associations between CKD and a wide range of previously described risk factors.

# Study Design

## General Design

This is a long-term longitudinal observational cohort study.

Patients will be followed for 10 years. Clinical assessments will take place as described at baseline, 1 year and 5 years. After this patients will no longer be assessed clinically but outcome data with respect to the primary end points as well as survival will be collected. The Medical Research Information Service (MRIS) will (an arm of the NHS Information Centre), will be used to accurately track survival data of participants.

## Primary Study Endpoints

### The primary end-point for assessing progression of CKD will be doubling of serum creatinine or the need for renal replacement therapy from baseline to 5 years and 10 years.

### The primary end-point for assessing cardiovascular risk will be a cardiovascular event, defined as myocardial infarction of any type (fatal and non-fatal, NSTEMI and STEMI), need for coronary revascularisation, TIA, fatal and non-fatal CVA, need for peripheral revascularization, amputation for peripheral vascular disease, death due primarily to peripheral vascular disease or sudden cardiac death at any time during 5 and 10 years of follow-up.

## Secondary Study Endpoints

### GFR value at baseline, 1, 5 and 10 years.

### Urine protein to creatinine ratio at baseline, 1, 5 and 10 years.

### Blood pressure at baseline, 1, 5 and 10 years.

### All-cause mortality over 5 and 10 years.

# Methods

## Subject Selection and Withdrawal

**Inclusion Criteria**

### Age >18 years

- CKD stage 3 as defined by the K/DOQI classification (i.e. GFR 30-59ml/min/1.73m^2^ on 2 occasions at least 3 months apart)

**Exclusion Criteria**

- Inability to attend GP surgery
- Inability to provide informed consent

## Subject Recruitment and Screening

Patients with CKD stage 3 (as defined above) will be identified from General Practice CKD registers and biochemistry databases by participating General Practitioners. GPs will send out the patient information sheet and a letter of invitation to potential participants. Patients will be invited to set up an appointment for the initial assessment by telephoning a number at Royal Derby Hospital. If patients do not wish to participate they will be asked to confirm this by telephoning the same number. If no response has been received within 2 weeks a second letter may be sent.

Informed consent to participate in the study will be obtained at the time of the first visit by the researchers, this will include consent to certain identifiable information being passed on to the Medical Research Information Service. Patients will have at least 1 week to decide about participation.

No additional screening procedures will be required prior to entry.

Each patient will be assigned a unique study number that will be used to identify all data collected for the study. This will be allocated at the first study visit. A key of patient identity and study numbers will be kept on a password-protected Trust computer in a password-protected file. Any information passed onto the Medical Research Information Service will be in encrypted form.

A recruitment log will be maintained.

Details of participation in the study will be recorded in the patients’ GP records.

## Withdrawal of Subjects

Patients will be withdrawn only if they withdraw their consent.

### When and How to Withdraw Subjects

Patients who withdraw their consent will be removed from the study immediately. They will continue to receive routine care through their GP.

### Data Collection and Follow-up for Withdrawn Subjects

Patients who withdraw will be asked to allow data collected prior to the date of withdrawal to be retained for analysis. They will also be asked to allow outcome data, including clinical events and the results of any kidney function tests arranged by the GP, to be collected for future analysis. If a participant declines this their data will be deleted.

Patients who fail to attend for follow-up will be contacted by telephone to determine whether or not they are willing to continue. Patients without access to a telephone will be contacted by letter. If no response is received to two letters sent one month apart, the patient will be assumed to have been lost to follow-up.

## Prior and Concomitant Therapy

All current medications will be recorded. Patients will be asked if they have previously been treated with non-steroidal anti-inflammatory drugs, angiotensin-converting enzyme inhibitors or angiotensin receptor blockers for a period of 3 months or longer.

# Laboratory Assays

The following laboratory tests will be obtained at baseline, 1 year and 5 years. The majority of these tests form part of routine clinical care and would normally be performed annually by the General Practitioner. Tests marked with an asterix are not routine and will be performed by the researcher in a research laboratory. Participants will be asked not to eat meat for 12 hours prior to the blood tests (to avoid a potential effect on the creatinine assay).

- Urea and electrolytes
- Estimated GFR
- Calcium and phosphate
- Serum albumin
- Lipid profile
- Random blood glucose
- Bicarbonate
- Uric acid
- Full blood count
- Urine albumin:creatinine ratio (on three early morning specimens)
- Urine protein:creatinine ratio (on three early morning specimens)
- Urine sodium (on three early morning specimens)
- Urine phosphate (on three early morning specimens)
- 24h urine collection for measurement of urinary creatinine, protein, albumin, sodium and phosphate will be performed in a subgroup of 160 patients.
- High sensitivity C-reactive protein (CRP)
- ADMA (asymmetrical dimethyl arginine)
- Serum fibroblast growth factor 23 (FGF 23)*
- Serum 25 hydroxy-vitamin D
- Serum 1,25 dihydroxy-vitamin D
- Serum Parathyroid hormone
- Serum Cystatin C
- Serum free light chains
- Serum will be stored for future biochemical assays but these will not include genetic testing. This to allow testing of substances that may be identified as significant risk factors for renal or cardiovascular outcomes during the follow-up period. Serum will not be stored beyond the duration of the study.

# Randomisation

No applicable.

# Study Procedures

- Patients will be asked to complete an information sheet to provide demographic and medical history details prior to attending for the first study assessment. They will also be asked to complete a brief food frequency questionnaire to assess salt intake. Demographic and medical history will be assessed only at baseline. Current medication will be assessed at each study visit. The food frequency questionnaire will be repeated at each study visit. Medical history and treatment details will be checked against the GP records. If known, the cause of the CKD will be recorded and assigned a corresponding European Dialysis and Transplantation Association diagnostic code.
- The first part of the assessment (5-10 minutes) will comprise a patient interview to clarify details and complete any unfilled sections on the information sheet and food frequency questionnaire.
- A measure of socio-economic status with be derived from participants’ post code (Indicies of Multiple Deprivation(IMD) score)
- Quality of life will be assessed using the EQ5D questionnaire
- Functional status will be graded using the Karnofsky score
- Anthropomorphic Measurements (repeated at each study visit)
  - Height
  - Weight
  - Body Mass Index
  - Hip to waist ratio
  - Mid-upper arm muscle circumference
  - Hand grip strength
  - Gait speed
  - Sit to stand test
  - Blood pressure (seated; average of 3 readings recorded with an oscillometric device)
  - Peridontal assessment
  - Urine dipstick test
  - Non-invasive assessment of arterial pulsewave velocity (Viacorder, Skidmore Medical Ltd, Bristol, UK)
  - Non-invasive measurement of forearm skin autofluoresence as a measure of advanced glycation end product deposition (AGE Reader, DiagnOptics, Groningen, Netherlands)
- Venesection (repeated at each study visit)
  - 3 x 5ml clotted blood
  - 1x 3ml blood in EDTA
- Urine collection (repeated at each study visit)
  - 3 x 5-10ml first morning urine specimen

Each study visit is expected to last 30-40 minutes.

# Statistical Plan

Statistical advice has been sought from Derby Hospitals NHS Foundation Trust Statistician, Mr. Apostolos Fakis.

## Sample Size Determination

The sample size was estimated based on two similar studies, one for predicting renal outcome in IgA nephropathy [4] and the other in patients with type 2 diabetes and nephropathy [3]. Based on these studies the smaller event rate was 12% and the smallest significant Hazard Ratio was 1.2. Therefore the total sample size for this study, using the Cox Proportional Hazards models with Power 80% and at significant level 5%, is 1968. Assuming death rate during the 5 years of 3% and drop out rate of 10% the final total sample is 2255. We plan to complete recruitment in 1 year and therefore aim to recruit approximately 190 participants per month. CKD stage 3 is estimated to affect at least 5% of the UK population. There should therefore be approximately 35,000 patients available for recruitment in Derbyshire.

A separate sample size estimate was performed for the sub-study comparing spot early morning urine biochemistry with that from 24hour urine collections. Based on previously published data, we calculated that a sample of 160 would allow us to show a correlation co-efficient of 0.9 with precision of the estimate of ±0.03 at 80% power with a significance of 5% [7].

## Statistical Methods

Basic demographic data will be retained on the anticipated small number of excluded patients as these form part of the at risk population. Descriptive statistics will be used to provide a summary of each of the variables collected at each time point. Multivariable linear regression analysis will be used to assess the effect of baseline variables as well as changes in these variables over the first 12 months on changes in blood pressure, proteinuria and glomerular filtration rate. Cox proportional hazards models will be used to identify independent baseline risk factors for CKD progression and cardiovascular events at 5 and 10 years. A similar method will be used to assess the possibility that changes in baseline variables from baseline to 12 or 24 months may be better predictors of CKD progression and cardiovascular events than baseline variables alone. Coefficients derived from Cox proportional hazards analysis for the independent determinants of CKD progression or cardiovascular events will then be used to produce a risk scoring system.

In a subgroup of 160 patients, assessment of proteinuria by urine protein to creatinine ratio versus albumin to creatinine ratio or 24h urinary protein will be assessed. Intraclass Correlation Coefficients will be used for comparing the three methods and Bland-Altman plots will be performed. 24 hour urinary protein will be the standard against which the other methods are compared. Similar analysis will be performed to compare urinary sodium and phosphate assays in spot and 24 hour urine specimens.

Estimated GFR values obtained with a new creatinine-cystatin-C equation [12] will be compared to eGFR derived from creatinine alone using a paired t-test to compare the mean values and by Bland-Altman plots. The intraclass correlation coefficients (95% Confidence Interval) will be used to check the agreement of eGFR estimates by different equations. The analysis will be repeated in clinically important subgroups including participants under 75 years versus 75 years or older, males versus females and those with diabetes versus those without. The change in eGFR over 5 years determined by each of the GFR estimating equations will be similarly compared. We will compare the performance of prognostic models using eGFR derived from creatinine versus combined creatinine-cystatin-C equation with respect to discrimination (C-statistic derived from ROC curves) and calibration (Hosmer Lemeshow test, net reclassification index (NRI)). We will repeat these analyses using change in GFR (estimated from creatinine or combined equations) and albuminuria over the first year of follow-up as independent variables as well as baseline values to test whether change over the first year adds to the prognostic model.

## Subject Population(s) for Analysis

All patients will be included in the analyses described above except for the analyses that include results derived from 24 hour urine collections (subgroup of 160 patients only). Data from patients who withdraw (and give consent) or who die will be included until the date of withdrawal or death. Analyses of data from 24 hour urine collections will be applied only to the relevant subgroup.

# Safety and Adverse Events

## Recording of Adverse Events

All Adverse Events (AE) Serious Adverse Events (SAE) will be recorded according to the R&D Office Procedure and the Trust Policy on Incident Reporting.

## When Adverse Events are Recorded

At each contact with a participant, the researcher will seek information on adverse events by specific questioning and by examination if appropriate. Information on all adverse events will be recorded immediately in the source document, and also in the appropriate Adverse Event Report form. If the event fulfils the requirement of an IR1, it will also be reported according to Trust Policy. Adverse events will also be reported to the participant’s GP. All SAEs will be reported to the Sponsor (Derby Hospitals NHS Foundation Trust) immediately and to the Research Ethics Committee according to REC requirements

All adverse events occurring during the study period will be recorded. The clinical course of each event will be followed until resolution, stabilization, or until it has been determined that the study treatment or participation is not the cause. Serious adverse events that are still ongoing at the end of the study period will be followed up to determine the final outcome.

As this is an observational study, it is not anticipated that there will be any adverse events directly related to the study. Nevertheless it is possible that the study will identify adverse events related to drugs prescribed by the GP. Venesection may be associated with minor discomfort but all other procedures are non-invasive and not associated with adverse effects. Death as well as the outcomes described in the primary end-points are expected events during the 10 years of follow-up.

# Data Handling and Record Keeping

## Confidentiality

Information about study subjects will be kept confidential and managed according to the requirements of the Data Protection Act, NHS Calldicott Guardian, The Research Governance Framework for Health and Social Care, Ethics Committee Approval and Trust IM&T Policy. Any information given as required for participant health status tracking, to the MRIS will be delivered in encrypted form.

Patient contact details including name, address and telephone number will be collected. This information will be retained to facilitate contacting the participants to arrange follow-up visits and tests. Only the researcher and an administrative assistant will have access to this database. It will be stored on a Trust password-protected computer. A single hard copy will be stored in a locked filing cabinet in the data custodian’s office.

Each participant will be assigned a unique study number. The patient’s name, date of birth and study number will be stored in a single password protected file on a Trust laptop. Only the researcher and principle investigator will have access to this file. It will be necessary for the researcher to take this file out of the Trust when seeing patients in order to verify their identity. All data collected for the study will be entered into a separate database in which data will be labeled only with the study number. The participant’s date of birth will be entered into this database but in the absence of other identifiable details in is unlikely that the participant could be identified from date of birth alone. Participants will not be identifiable from any publications arising from this study.

The Trust has a comprehensive registration with the Information Commissioner with research being registered under Purpose 4 of the Register; The Trust Data Protection Officer will be notified of all research studies in order to comply with the Trust IM&T Security Policy.

Dr Taal will act as “Custodian” of the data.

## Source Documents

Source documents for this study will include:

- Participant demographic and medical data sheet
- Food frequency questionnaire
- EQ5D questionnaire
- General Practitioner records
- Laboratory results reports
- Blood pressure, pulse wave velocity and AGE-Reader records

## Case Report Forms

Each participant will have a data collection form for each study assessment, labeled only with the unique study number. These will be completed in accordance with the principles of Good Clinical Practice.

## Records Retention

All paper records will be stored in a locked filing cabinet within the Trust or Medical School for the duration of the study. Computer records will be stored in password-protected files on password-protected computers in the Trust or Medical School. At the conclusion of the study all data will be archived and stored for 10 years in a secure storage facility in accordance with Trust policy.

Patient participation will be recorded in the GP medical records.

Three copies of the consent from will be obtained. One will be given to the participant and the others will be filed in the participant’s GP records and with the data collection form, respectively.

# Study Monitoring, Auditing, and Inspecting

## Study Monitoring Plan

The investigator will permit study-related monitoring, audits and inspections by the Ethics Committee, the Sponsor and the Research Governance Manager. This study will be monitored by the Research Governance Manager according to the Research & Development Office procedure for monitoring all non-commercial research carried out at the Derby Hospitals NHS Foundation Trust and the Southern Derbyshire Primary Care Trusts. In line with the responsibilities set out in the Research Governance Framework, the Investigator will ensure that the research governance manager or other regulatory monitoring authority is given access to all study-related documents and study related facilities.

# Ethical Considerations

This study will be conducted according to the standards of International Conference on Harmonization, Good Clinical Practice Guideline, Research Ethics Committee regulations, any applicable government regulations, Trust and Research Office policies and procedures.

This protocol and any amendments will be submitted to a properly constituted Research Ethics Committee (REC) for approval of the study conduct.

All subjects for this study will be provided with an information sheet describing the elements of this study and sufficient information for subjects to make an informed decision about their participation in this study. See Attachments for a copy of the Information Sheet. The subject will complete and sign a consent form to indicate that they are giving valid consent to participate.

As this is an observational study it is not anticipated that any ethical problems will arise. Children and vulnerable adults will not be included.

# Study Finances

## Funding Source

The study was funded initially through an unrestricted educational grant from Roche Pharmaceutical Products. Additional funding was obtained in the form of a Research Fellowship from the British Renal Society and Kidney Research UK as well as Genzyme (now part of Sanofi). Neither of the companies providing funding had any involvement in the design or conduct of the study nor do they have any right to the study data. Funding for the year-5 follow-up phase of the study has been obtained from The Dunhill Medical Trust.

## Indemnity for the performance of the study

NHS indemnity will apply in the event of a claim by, or on behalf of, participants for negligent harm. There will be no special arrangements for non-negligent harm but the normal NHS Complaints mechanism will be available to all participants.

## Sponsorship:

Sponsorship will be arranged between the chief investigator and Derby Hospitals NHS Trust.

# Publication Plan

Dr Taal will have primary responsibility for publication of the results. It is intended that the results will be presented at national and international nephrology conferences and published in high quality nephrology journals. The results will also be presented in a dissertation for a PhD degree.

# References

1. Taal MW, Brenner BM. Predicting initiation and progression of chronic kidney disease: Developing renal risk scores. *Kidney Int* 2006; **70**:1694-1705.

2. Kent DM, Jafar TH, Hayward RA, et al. Progression Risk, Urinary Protein Excretion, and Treatment Effects of Angiotensin-Converting Enzyme Inhibitors in Nondiabetic Kidney Disease. *J Am Soc Nephrol*. 2007; **18**:1959-1965.

3. Keane WF, Zhang Z, Lyle PA, et al. Risk scores for predicting outcomes in patients with type 2 diabetes and nephropathy: The RENAAL Study. *Clin J Am Soc Nephrol* 2006; **1**:761-767.

4. Wakai K, Kawamura T, Endoh M, et al. A scoring system to predict renal outcome in IgA nephropathy: from a nationwide prospective study. *Nephrol Dial Transplant.* 2006; **21**:2800-2808.

5. Go AS, Chertow GM, Fan D, et al. Chronic kidney disease and the risks of death, cardiovascular events, and hospitalization. *New Engl J Med* 2004; **351**:1296-1305.

6. Meerwaldt R, Hartog JW, Graaff R, et al. Skin autofluorescence, a measure of cumulative metabolic stress and advanced glycation end products, predicts mortality in hemodialysis patients. *J Am Soc Nephrol* 2005; **16**:3687-3693.

7. Fisher MA, Taylor GW, Brent JS, *et al*. Periodontal disease and other nontraditional risk factors for CKD. *Am J Kidney Dis* 2007; **51**: 45-52.

8. Wu T, Trevisan M, Grenco RJ, *et al*. Periodontal disease and risk of cerebro-vascular disease. *Arch Intern Med* 2000; **160**: 2749-2755.

9. Dietrich T, Jimenez M, Kaye EA, *et al*. Age-dependant associations between chronic periodontitis/edentulism and risk of coronary heart disease. *Circulation* 2008; **117**: 1668-1674.

10. Shultis WA, Weil EJ, Looker HC *et al*. Effect of periodontitis in overt nephropathy and end stage renal disease in type 2 diabetes. *Diabetes Care* 2007; **30(2)**: 306-311.

1. Ruggenenti P, Gaspari F, Perna A, *et al*. Cross sectional longitudinal study of spot morning urine protein:creatinine ratio, 24 hour urine protein excretion rate, glomerular filtration rate, and end stage renal failure in chronic renal disease in patients without diabetes. *BMJ* 1998; **316:**504-509.
2. Inker LA, Schmid CH, Tighiouart H, Eckfeldt JH, Feldman HI, Greene T, Kusek JW, Manzi J, Van Lente F, Zhang YL, Coresh J, Levey AS. Estimating glomerular filtration rate from serum creatinine and cystatin C. N Engl J Med 2012; 367:20-29.
