# Supplementary material for: The association of skin autofluorescence with cardiovascular events and all-cause mortality in persons with chronic kidney disease stage 3: A prospective cohort study
Source: PLoS Med. 2020 Jul 13;17(7):e1003163. doi: 10.1371/journal.pmed.1003163 (PMC7357739; doi:10.1371/journal.pmed.1003163)
Supplement: S1 Questionnaire — (DOCX) [file pmed.1003163.s010.docx]

**BACKGROUND INFORMATION FORM**

**Defining the risk of renal function decline and cardiovascular disease among patients with chronic kidney disease stage 3: The Renal Risk in Derby (R^2^ID) Study.**

**STUDY NUMBER: ………………………DATE OF VISIT……………………….**

**VISIT YEAR (circle as appropriate): Baseline 1 2 5**

Please complete each of the questions below. If you are uncertain, leave the section blank and we will help you to complete it at your first study visit.

1. Date of birth (dd/mm/yyyy): ___/___/______

2. Gender: (please circle) Male / Female

3. Ethnicity: (please circle) White / Mixed / Asian / African-Caribbean / Chinese or Other (please state): ………………………….

4. Highest Qualification: (please tick just highest level achieved)

None GCSE (or equivalent) A levels

NVQ levels 1-3 NVQ levels 4-5 (or equivalent) First Degree

Higher Degree

5. Do you have a close relative (those listed below) with kidney disease? YES / NO

If “YES” please circle which: father / mother / brother / sister / child

6. Do you have a close relative who currently receives or previously received dialysis treatment or a kidney transplant? YES / NO

If “YES” please circle which: father / mother / brother / sister / child

7. Do you have a close relative with diabetes? YES / NO

If “YES” please circle which: father / mother / brother / sister / child

8. Please record your birthweight: ……pounds ……ounces

9. Are you currently or have you previously been affected by any of the following (please tick if yes):

Diabetes 🞏

Angina 🞏

Myocardial Infarction (Heart Attack) 🞏

Heart Failure 🞏

Stroke 🞏

Transient Ischaemic Attack (Mini-stroke) 🞏

Narrowed arteries in your limbs 🞏

Amputation of a limb 🞏

10. **Ladies only**: Have you previously been pregnant? YES / NO

If “yes”, were you affected by any of the following during your pregnancy (please tick if yes):

High Blood Pressure 🞏

Protein in the urine 🞏

Kidney Failure 🞏

Seizures 🞏

11. Have you been admitted to hospital in the past year? YES / NO

If “yes” was this an emergency admission? YES / NO

If “yes” how many days have you spent in hospital in the past year? ……..

If “yes” did you have antibiotics as an inpatient? YES / NO

12. Have you ever been seen by a Kidney (Renal) Consultant or Nephrologist? YES / NO

13. Are you still under the care of a Kidney (Renal) Consultant or Nephrologist? YES / NO

14. Have you ever had a kidney biopsy? YES / NO

15. Do you currently smoke cigarettes? YES / NO

If “yes”, for how many years have you smoked? ……….

If “yes”, how many cigarettes do you smoke per day? ……….

Have you ever tried to stop smoking? YES / NO

16. Have you previously smoked cigarettes? YES / NO

If “yes”, for how many years did you smoke? ……….

If “yes”, how many cigarettes did you smoke per day? ………..

When did you stop smoking? (year) ………..

17. Do you drink alcohol? YES / NO

If “yes” how many of the following do you have per week:

Pints of beer ……….

Pints of cider ……….

Glasses of red wine ……….

Glasses of white wine ……….

Tots of spirits ……….

Cocktails or other drinks ……….

18. Do you regularly take any of the following pain medications (please tick)?

Paracetamol 🞏

Co-codamol 🞏

Ibuprofen (Brufen/Nurofen) 🞏

If “yes”, how many tablets do you take per week? ……….

If “yes”, for how many years have you taken the pain medication?

19. Please write the names and doses of all the medication you are currently taking:

Drug name Dose

------------------------------------------------- --------

------------------------------------------------- --------

------------------------------------------------- --------

------------------------------------------------- --------

------------------------------------------------- --------

------------------------------------------------- --------

------------------------------------------------- --------

------------------------------------------------- --------

------------------------------------------------- --------

------------------------------------------------- --------

------------------------------------------------- --------

------------------------------------------------- --------

------------------------------------------------- --------

Thank you for taking time to complete this form.

**Please bring it with you to your study visit.**
