# Supplementary material for: The association of skin autofluorescence with cardiovascular events and all-cause mortality in persons with chronic kidney disease stage 3: A prospective cohort study
Source: PLoS Med. 2020 Jul 13;17(7):e1003163. doi: 10.1371/journal.pmed.1003163 (PMC7357739; doi:10.1371/journal.pmed.1003163)
Supplement: Attachment — Submitted filename: Requests from the editors.docx [file pmed.1003163.s011.docx]

**Responses to Editors’ and Reviewers’ Comments:**

Requests from the editors:

Title- Please revise your title according to PLOS Medicine's style. Your title must be nondeclarative and not a question. It should begin with main concept if possible. "Effect of" should be used only if causality can be inferred, i.e., for an RCT. Please place the study design ("A randomized controlled trial," "A retrospective study," "A modelling study," etc.) in the subtitle (ie, after a colon).

**Response:** The title has been changed in response to a comment from Reviewer 2 and to conform to PLOS Medicine’s style: “The association of skin autofluorescence, a measure of advanced glycation endproduct accumulation, with cardiovascular events and all-cause mortality in persons with chronic kidney disease stage 3: a prospective cohort study”

Abstract- please provide details of where this study was carried out, dates of patient recruitment/ data collection as appropriate and brief patient demographics

**Response:** We have added these additional details to the Abstract as requested.

Abstract methods and findings- the last sentence should be a limitation of your study design

**Response:** A sentence summarising the most important limitation has been added.

The Data Availability Statement (DAS) requires revision. For each data source used in your study:

a) If the data are freely or publicly available, note this and state the location of the data: within the paper, in Supporting Information files, or in a public repository (include the DOI or accession number).

b) If the data are owned by a third party but freely available upon request, please note this and state the owner of the data set and contact information for data requests (web or email address). Note that a study author cannot be the contact person for the data.

c) If the data are not freely available, please describe briefly the ethical, legal, or contractual restriction that prevents you from sharing it. Please also include an appropriate contact (web or email address) for inquiries (again, this cannot be a study author).

**Response:** The Data Availability Statement has been amended as follows:

Anonymised data can be made available to researchers who meet the conditions of the ethics approval and research governance policy that applies to this study. Researchers may apply for data access by contacting Dr. Teresa Grieve, Research and Development Deputy Director, University Hospitals of Derby and Burton NHS Foundation Trust (teresa.grieve@nhs.net).

At this stage, we ask that you include a short, non-technical Author Summary of your research to make findings accessible to a wide audience that includes both scientists and non-scientists. The Author Summary should immediately follow the Abstract in your revised manuscript. This text is subject to editorial change and should be distinct from the scientific abstract. Please see our author guidelines for more information: <https://journals.plos.org/plosmedicine/s/revising-your-manuscript#loc-author-summary>

**Response:** An Author Summary has been added after the Abstract

STROBE checklist-please do not use page numbers as these are likely to change. Please use paragraphs and sections instead

**Response:** This has been done as requested.

Please add the following statement, or similar, to the Methods: "This study is reported as per the Strengthening the Reporting of Observational Studies in Epidemiology (STROBE) guideline (S1 Checklist)."

**Response:** This has been added as requested.

Questionnaires- please provide a copy of all questionnaires used in the study, if previously published please provide a citation. The same goes for the equations mentioned on Pages 6 and 7

**Response:** We used a questionnaire to facilitate data collection. This has been added to the Supplementary material. The MDRD and CKD-EPI equations used have been published and are in widespread use by chemistry laboratories globally. Rather that providing the equations we have provided references for the primary publications. I would be willing to include the equations but I don’t think this would add anything to the paper.

Did your study have a prospective protocol or analysis plan? Please state this (either way) early in the Methods section. a) If a prospective analysis plan (from your funding proposal, IRB or other ethics committee submission, study protocol, or other planning document written before analyzing the data) was used in designing the study, please include the relevant prospectively written document with your revised manuscript as a Supporting Information file to be published alongside your study, and cite it in the Methods section. A legend for this file should be included at the end of your manuscript. b) If no such document exists, please make sure that the Methods section transparently describes when analyses were planned, and when/why any data-driven changes to analyses took place. c) In either case, changes in the analysis-- including those made in response to peer review comments-- should be identified as such in the Methods section of the paper, with rationale.

**Response:** The study was conducted according to a prospective protocol which was approved by a research ethics committee. This has been clarified in the Methods section and the protocol has been added to the Supporting Information.

Please replace Caucasian with white

**Response:** This has been done.

Please include all financial information into the financial disclosure section within article meta-data instead of the acknowledgements

**Response:** This has been removed from the acknowledgements

Comments from the reviewers:

Reviewer #1: I confine my remarks to statistical aspects of this paper. The general approach is mostly fine, but I do have some issues to resolve before I can recommend publication.

Line 36 (and 162) "Determinants" is too causal. I'm not sure what word to suggest here. Perhaps the editors have a suggestion. But, this is an observational study , so, we only know that these were associated with CVE and ACM.

**Response:** We have removed the term “determinants” from the manuscript and replaced this with “variables associated with” throughout.

Line 38	What is after the +- sign? Is that an SD? A 95% CI? or something else?

**Response:** As assumed by the reviewer, the number after the “±” is a standard deviation. This was originally stated in the “Statistical Methods” paragraph and footnote to Table 1 but we have clarified it in the abstract.

Line 141 - not a stats comment, but maybe say whether it was the posterior or anterior portion of the forearm? (I only mention this because you discuss skin color and the anterior portion is differently colored than the posterior)

**Response:** SAF was measured on the ventral (anterior) surface of the forearm. This has been clarified in the methods.

Line 158 - Please specify that it is tertiles of SAF. But why compare across tertiles using ANOVA? it would be better to leave SAF continuous and use regression. (Tertiles might be useful for a table, but categorizing variables for analysis loses power. In addition, nonlinearities can be investigated with splines.

**Response:** The original manuscript states in the first paragraph of the results “Baseline characteristics are presented in Table 1 by tertile of SAF.” This is also stated in the legend for Table 1. We have presented linear correlations between baseline variables and baseline SAF in a previous paper. In this paper we chose to present the data divided according to tertiles of baseline SAF to give the reader a more concrete impression of the magnitude of change in clinical variables with increasing SAF. The use of tertiles also corresponds with the Kaplan Meier analyses.

Line 169-170 Linear regression on change scores is not generally recommended, unless the scores are measured perfectly - I'm not sure how well SAF measurements work - a better method is a multilevel model.

**Response:** We thank the reviewer for this helpful comment. We have obtained additional advice and assistance from a statistician and have constructed multi-level mixed effects models to assess factors associated with change in SAF over the 5 year follow-up period. These results are presented in a new Table 2. There were some relatively minor changes in the associations compared with the previous linear regression analysis. The results and discussion have been updated to reflect these changes.

Line 170-172 Why were variables log transformed? Linear regression makes no assumptions about the distribution of the variables (it makes assumptions about the residuals).

**Response:** We have used now used the original variables in the multi-level modelling. Log transformed values were used in the Cox regression models to allow expression of the hazard ratios per standard deviation change in each independent variable

Figures 2 and 3 - I would use "Years" rather than "Days" just for ease of reading. (People don't think "400 days" they think "a year and a quarter"). I would consider limiting the y axis to 0.5 and up - although this is debatable. It allows finer discrimination but might give the wrong idea to someone who doesn't look at the y axis.

**Response:** We have changed the time scale on the x-axis to years as suggested. We would prefer to leave the y-axis unchanged for the reason that this appears to exaggerate the true effect and may mislead a casual reader.

Peter Flom

Reviewer #2: The authors analyze the associations of skin autofluorescence (SAF), a biomarker of tissue accumulation of advanced glycation endproducts, with incidence of cardiovascular events and mortality, in a cohort of 1707 patients affected by stage 3 chronic kidney disease. SAF is a non invasive and quick measurement, which appears to be predictive of adverse health outcomes in particular in diabetic patients. Several studies have evidenced an association of SAF with increased risk for cardiovascular disease and mortality in patients in hemodialysis. However, there are few available data on this association at earlier stages of CKD, who exhibit higher SAF values than the general population. This study thus addresses an important issue, which could help identify specific mechanisms of cardiovascular disease in CKD patients. The methods are sound and the paper is generally well written.

In addition to the analysis of incidence of cardiovascular events and all-cause mortality, the authors should consider adding supplementary analyses regarding **cardiovascular and non-cardiovascular mortality**. Indeed, it would be informative to differentiate the associations of SAF with **fatal versus non fatal cardiovascular events**, as well as with mortality from other causes, in order to document the specificity of these associations.

**Response:** We have conducted these supplementary analyses as suggested and the data are presented in three additional Supplementary Tables. Owing to the lower number of events, the number of variables included had to be reduced for the analysis of cardiovascular deaths. Furthermore, these supplementary analyses should be interpreted with some caution due to reduced statistical power resulting from a lower number of events in each subgroup.

Specific comments:

Title: As "skin autofluorescence" is a rather unspecific term, consider adding "advanced glycation endproducts" in the title.

**Response:** This has been done as suggested.

Abstract: add the number of participants in the methods.

**Response:** This has been done

Statistical methods (page 8, lines 162-168): in the Cox models, SAF was modeled as a continuous variable, thus hypothesizing a linear relationship of SAF with the outcomes. Has this hypothesis been verified ?

**Response:** A linear relationship between SAF and the outcomes was confirmed by inspection of the Kaplan-Meier plots (Figures 2 and 3)

Table 1: there is one decimal missing for phosphate in the lowest tertile. A second decimal could also be added for this variable.

**Response:** Thank you for pointing this out. It has been corrected.

Tables 3 and 4: were there any missing data for the covariables ? Please add number of analyzed participants in all analyses.

**Response:** Details regarding missing variables are shown in Table 1. The number of participants included in each model has been added to the other Tables.

Discussion (page 21): the authors should also cite the potential selection bias as a limitation of this study, as only 1741 of 8280 invited patients were initially included. This may limit the generalization of the results.

**Response:** This has been done.

Reviewer #3: This is an important prospective study in a large cohort of patients at an early stage of Chronic Kidney Disease regarding skin autofluorescence as a risk factor for cardiovascular events and all-cause mortality.

Increased cardiovascular morbidity and mortality in CKD patients is caused by traditional and non-traditional risk factors. The search for new non-traditional CV risk factors, particularly modifiable, which may affect the prognosis in this group of patients, is an important clinical aspect of the study.

My suggestions:

Methods:

1.	What kits were used to analyze urine albumin to creatinine ratio?

**Response:** Details of the assay used for urinary albumin have been added to the Methods section.

2.	As the medication history was collected at each visit please include the information on how many patients were on lipid lowering therapy?

**Response:** We unfortunately did not have complete data on lipid lowering therapy and this was therefore not included in the analysis.

3.	How many patients had malnutrition?

**Response:** Formal nutritional assessment was not included in the protocol and data were therefore not available for analysis.

4.	Both total and HDL cholesterol were used in analyses. Why was the LDL cholesterol, which increased concentration is a known CV mortality risk factor, and is used as therapeutic goal, not a part of the analyses?

**Response:** Total cholesterol and HDL cholesterol were directly measured in the laboratory but LDL cholesterol was not. We therefore chose to use directly measured rather than derived variables.

5.	Line 169 - BMI abbreviation has not been previously expanded

**Response:** This has been corrected

Results:

1. Line 184 - SBP abbreviation has not been previously expanded

**Response:** This has been corrected

2. Line 185 - DBP abbreviation has not been previously expanded

**Response:** This has been corrected

Discussion:

1. The Authors properly identify that "the association with lower cholesterol may have resulted from reverse causality due to more persons with CVD receiving lipid lowering therapy." However, the most commonly used lipid lowering drugs, statins, except for lowering cholesterol concentrations, possess pleiotropic effects, including decreasing RAGE expression. Therefore statin taking, as a potential confounder, should be a part of the analyses.

**Response:** We agree that consideration of lipid lowering therapy as a potential confounder would have been helpful but unfortunately we do not have data on lipid lowering medication. We have clarified this in the discussion.

2. Please include in limitations that office BP and not ABPM was measured. In CKD patients specific form of hypertension - masked hypertension and non-dipping BP profile, which are known to increase CV risk, are present. The diagnosis is possible only with the use of ABPM.

**Response:** We have added discussion of these issues to the limitations section.

Acknowledgements:

1.	According to PLOS submission guidelines please "Do not include funding sources in the Acknowledgments or anywhere else in the manuscript file. Funding information should only be entered in the financial disclosure section of the submission system."

**Response:**

Reviewer #4: This is a well designed prospective study performed by an experienced team in the field. In fact, it is a continuation of an already published work by these authors. It adds on additional information of the role of AGEs on the clinical outcome of the patients with Chronic kidney disease stage 3. There is also some new information in this paper: the finding that over time the accumulation of AGEs in the body rises, with an implication that the risk for development of cardiovascular events or all cause mortality rises too. It also points out the direction of future research in this field, designing prospective interventional clinical studies with an intention to reduce AGEs and consequently the adverse clinical outcomes in the patients. The paper is clearly and critically written. It is a significant contribution to the existing literature. I would like to congratulate the authors for the excellent work and gladly recommend publication.

**Response:** We sincerely thank the reviewer for these very supportive comments
