# Supplementary material for: The association of skin autofluorescence with cardiovascular events and all-cause mortality in persons with chronic kidney disease stage 3: A prospective cohort study
Source: PLoS Med. 2020 Jul 13;17(7):e1003163. doi: 10.1371/journal.pmed.1003163 (PMC7357739; doi:10.1371/journal.pmed.1003163)
Supplement: Attachment — Submitted filename: Response to Editors Comments.docx [file pmed.1003163.s012.docx]

**Response to Editor’s and Reviewers’ Comments**

**The association of skin autofluorescence with cardiovascular events and all-cause mortality in persons with chronic kidney disease stage 3: a prospective cohort study** (PMEDICINE-D-20-00405R3)

**Requests from Editors:**

Title- Consider shortening to “The association of skin autofluorescence with cardiovascular events and all-cause mortality in persons with chronic kidney disease stage 3: a prospective cohort study”

**Response:** The title has been changed as suggested. It is now the same as the original title which was changed in response to a Reviewer’s comment.

Abstract

Please provide brief participant demographics

**Response:** The previous version of the abstract did report mean age and percentage females. I have added the percentage of participants with white ethnicity. I’ve also added these data to the first paragraph of the Results section in the paper.

Last sentence of methods and findings must state a limitation of your study design/methodology

**Response:** I have added a sentence discussing a limitation of the methodology used.

Competing Interests- please include a note to state that Maarten Taal is an Academic Editor at PLOS medicine

**Response:** My apologies, I thought I had done this. It has been added.

Data availability- please can you update the meta-data section with the statement provided in the response to comments? It seems the original data statement was not updated, we would be grateful if you could change this.

**Response:** The data availability statement has been updated in the meta-data section to: Anonymised data can be made available to researchers who meet the conditions of the ethics approval and research governance policy that applies to this study. Researchers may apply for data access by contacting Dr. Teresa Grieve, Research and Development Deputy Director, University Hospitals of Derby and Burton NHS Foundation Trust (teresa.grieve@nhs.net).

Author Summary

Would it be possible to combine the last 3 points in the “why was this done” section to two bullet points? Perhaps combine the third and fourth points?

**Response:** This has been done.

In the “what did the researchers do and find” section, we only need the key messages. I suggest combining the second and third point. The last point can perhaps be removed since you mention this again in the “what do these findings mean” section.

**Response:** I have combined the first two bullet points and removed the last point.

Results

Line 235- please replace “diabetic” with “with Type 1/2 diabetes”

**Response:** This has been changed as requested. I think it was line 253.

Please format the bibliography using Vancouver style

**Response:** The bibliography has been formatted using Vancouver style.

Individual items in the supplementary information like the STROBE checklist, study protocol and questionnaires etc will need to be dissociated and uploaded as individual files.

**Response:** The supplementary information has been placed in separate files as requested.

**Comments from Reviewers:**

Reviewer #1: The authors have addressed my concerns and I now recommend publication

Peter Flom

Reviewer #2: The authors have adequately answered most of the reviewers' comments. I have only two additional comments:

- I don't think that Figures 2 and 3 allow to correctly assess the linearity of the association of SAF with ACM and CVE. I think that the authors should test this hypothesis, for instance by using splines as previously suggested by reviewer #1.

**Response:** We consider that we have adequately addressed the original comment. We can find no record that Reviewer #1 suggested doing additional analysis using splines. We therefore propose not to do this additional analysis unless requested by the Editors.

- Regarding cholesterol, I understand that LDL cholesterol can only be deducted from total and HDL-cholesterol. However, as total includes HDL-cholesterol, I think that it would be more correct to analyse HDL- and non HDL-cholesterol, rather than HDL and total cholesterol (also because non HDL cholesterol is one of the strongest biomarker of cardiovascular disease in the general population as shown for instance by Brunner FJ et al. Lancet 2019 394(10215):2173-2183). I admit that this is a detail, since cholesterol does not appear to be associated with CVE or ACM in these CKD patients.

**Response:** The Reviewer has stated that this is a (minor) detail. To address this point would require that almost all the analyses are redone, which is not possible within the 1 week given for resubmission. We therefore propose not to do this additional analysis unless requested by the Editors.

Reviewer #3: I would like to congratulate the authors for the excellent work.

Reviewer #4: I examined the revised version. Earlier, I recommended accept. This version looks fine.

**Response:** We thank all the Reviewers for their supportive comments on the revised manuscript.
